# Application of the Box–Behnken Design in the Development of Amorphous PVP K30–Phosphatidylcholine Dispersions for the Co-Delivery of Curcumin and Hesperetin Prepared by Hot-Melt Extrusion

**DOI:** 10.3390/pharmaceutics17010026

**Published:** 2024-12-27

**Authors:** Kamil Wdowiak, Lidia Tajber, Andrzej Miklaszewski, Judyta Cielecka-Piontek

**Affiliations:** 1 Department of Pharmacognosy and Biomaterials, Poznan University of Medical Sciences, 3 Rokietnicka St., 60-806 Poznan, Poland; kamil.wdowiak@student.ump.edu.pl; 2 School of Pharmacy and Pharmaceutical Sciences, Trinity College Dublin, University of Dublin, D02 PN40 Dublin, Ireland; ltajber@tcd.ie; 3 Faculty of Materials Engineering and Technical Physics, Institute of Materials Science and Engineering, Poznan University of Technology, 5 M. Skłodowska-Curie Square, 60-965 Poznan, Poland; andrzej.miklaszewski@put.poznan.pl

**Keywords:** curcumin, hesperetin, hot-melt extrusion, amorphous solid dispersion, solubility-enabling formulation, phospholipid, Box–Behnken design

## Abstract

**Background:** Curcumin and hesperetin are plant polyphenols known for their poor solubility. To address this limitation, we prepared amorphous PVP K30–phosphatidylcholine dispersions via hot-melt extrusion. **Methods:** This study aimed to evaluate the effects of the amounts of active ingredients and phosphatidylcholine, as well as the process temperature, on the performance of the dispersions. A Box–Behnken design was employed to assess these factors. Solid-state characterization and biopharmaceutical studies were then conducted. X-ray powder diffraction (XRPD) was used to confirm the amorphous nature of the dispersions, while differential scanning calorimetry (DSC) provided insight into the miscibility of the systems. Fourier-transform infrared spectroscopy (FTIR) was employed to assess the intermolecular interactions. The apparent solubility and dissolution profiles of the systems were studied in phosphate buffer at pH 6.8. In vitro permeability across the gastrointestinal tract and blood–brain barrier was evaluated using the parallel artificial membrane permeability assay. **Results:** The quantities of polyphenols and phospholipids were identified as significant factors influencing the biopharmaceutical performance of the systems. Solid-state analysis confirmed the formation of amorphous dispersions and the development of interactions among components. Notably, a significant improvement in solubility was observed, with formulations exhibiting distinct release patterns for the active compounds. Furthermore, the in vitro permeability through the gastrointestinal tract and blood–brain barrier was enhanced. **Conclusions:** The findings suggest that amorphous PVP K30–phosphatidylcholine dispersions have the potential to improve the biopharmaceutical properties of curcumin and hesperetin.

## 1. Introduction

Curcumin is the main active ingredient found in the popular spice turmeric (*Curcuma longa* L.). This polyphenol is claimed to have a range of beneficial health-promoting properties that can be utilized in the treatment and prevention of chronic diseases. Curcumin is reported to exhibit antioxidant [1,2], anti-inflammatory [3,4], anticancer [5,6], antidiabetic [7,8], and neuroprotective [9,10,11] activities. Another promising polyphenol is hesperetin, a flavonoid found in citrus fruits. The compound has shown antioxidant [12], anti-inflammatory [13,14], anti-tumor [15,16], antidiabetic [17,18], and neuroprotective [19,20] effects. It is worth noting that co-administration of curcumin and hesperetin may provide synergistic neuroprotective effects by involving complementary mechanisms of action, thereby enhancing the effectiveness of treatment [21]. Research by Lee et al. showed that both curcumin and hesperetin exhibit significant antioxidant capabilities, which help reduce oxidative stress—a key factor in neurodegeneration—thereby protecting neuronal cells from damage. Moreover, the combination of these plant compounds modulated apoptotic pathways, decreasing the expression of pro-apoptotic proteins (like Bax) and increasing anti-apoptotic proteins (like Bcl-2), suggesting a protective effect against neuronal cell death. One study demonstrated that the combination of curcumin and hesperetin improved cognitive function in aged rats induced with D-galactose. The combined treatment showed notable effects, promoting neuron growth and reducing markers of cellular senescence [21]. However, both compounds suffer from low solubility, which limits their bioavailability and prevents them from demonstrating their full therapeutic potential. Many research groups have attempted to improve the bioavailability of these compounds. For curcumin fabrication of self-assembled cyclodextrin succinate/chitosan nanoparticles [22], the manufacturing of solid self-emulsifying systems [23] and production of pH-driven zein/tea saponin composite nanoparticles [24] have been reported. For hesperetin, techniques such as the creation of self-assembling rebaudioside A nanomicelles [25], nanoemulsions [26], and zein/pectin nanoparticles [27] have been developed.

One well-established technique for improving solubility is the conversion of the drug’s form from crystalline to amorphous. The amorphous form lacks long-range order, which results in increased solubility and dissolution rates compared to the crystalline form [28,29,30,31]. To prevent crystallization during dissolution and ensure physical stability, the addition of excipients such as polymers, is often necessary [32]. Another effective method for improving oral bioavailability is the use of phospholipids to prepare complexes with active substances [33,34,35]. Because of their ability to self-organize, micelles form during dissolution, which solubilizes the active compound [36]. Combining both mechanisms to enhance bioavailability may be a promising approach to formulation development.

This work aimed to develop amorphous polymer–phospholipid dispersions of curcumin and hesperetin to improve their biopharmaceutical potential. The goal of the research was achieved through several steps. Hot-melt extrusion technology was employed to produce the amorphous dispersions. Then, the Box–Behnken experimental design was selected to determine whether the amount of active substances, the quantity of phosphatidylcholine, and the temperature of the process significantly affected the solubility of the active compounds. The processed systems were subsequently subjected to solid-state characterization, followed by an evaluation of their biopharmaceutical properties. The novelty of this paper lies in the development of amorphous PVP K30–phosphatidylcholine dispersions aimed at increasing the solubility of poorly soluble plant compounds. Systems combining different solubility enhancement mechanisms—polymer and phospholipid dispersions—may represent an innovative solution for improving biopharmaceutical properties.

## 2. Materials and Methods

### 2.1. Materials

Hesperetin (purity > 95%) was sourced from Sigma-Aldrich (St. Louis, MO, USA), while curcumin (purity > 95%) was obtained from Xi’an Tian Guangyuan Biotech Co., Ltd. (Xi’an, China). The excipients were provided by the following manufacturers: PVP K30 and phosphatidylcholine (from dried egg yolk, purity 100%; CAS number: 8002-43-5; product number: 61771) from Sigma-Aldrich (St. Louis, MO, USA) and xylitol from Santini (Poznań, Poland). Other reagents included sodium hydroxide (Avantor Performance Materials Poland S.A., Gliwice, Poland), acetic acid (98–100%; POCH, Gliwice, Poland), sodium dimethyl sulfoxide (DMSO; Pan-Reac Appli-Chem ITW Reagents, Darmstadt, Germany), acetic acid (J. T. Baker, Center Valley, PA, USA), and HPLC-grade methanol (J. T. Baker, Center Valley, PA, USA). High-quality laboratory-grade water was produced using a Direct-Q 3 UV purification system (Millipore, Molsheim, France; model Exil SA 67120). The Prisma HT, GIT/BBB lipid solution, and acceptor sink buffer were supplied by Pion Inc. (Forest Row, East Sussex, UK).

### 2.2. Methods

#### 2.2.1. Preparation of PVP K30–Xylitol–(Phosphatidylcholine) Blends

PVP K30–phospholipid/PVP K30–xylitol blends were prepared by dissolving the required amounts of the ingredients to obtain 10 g of the blend in 100 mL of purified water (at 22 °C). To assess the plasticizing effect, blends with varying concentrations (*w*/*w*) of phospholipids and xylitol were made. Using the equations derived from the linear function, the amount of excipient needed to achieve a polymer–phospholipid–xylitol blend with a glass transition temperature of 115 °C was calculated (see Section 3). To prepare the PVP K30–phospholipid–xylitol mixture with a glass transition temperature of 115 °C, the excipients were weighed, transferred to a beaker, dissolved in 100 mL of purified water, and stirred using a magnetic stirrer. The resulting colloidal dispersion was then lyophilized (LyoQuest-85, Telstar, Terrassa, Spain) to produce a powder. The freeze-drying process was conducted at −82.1 °C under vacuum at a pressure of 0.412 mbar. The freeze-dried products were powdered using a mill (Tube Mill Control Mixer, IKA, Warsaw, Poland).

#### 2.2.2. Production of Extrudates

The hot-melt extrusion process was carried out using a HAAKE MiniCTW micro-conical twin-screw extruder (Thermo Scientific, Karlsruhe, Germany). PVP K30–phospholipid–xylitol or PVP K30–xylitol blends were mixed with 15%, 27.5%, or 40% active compounds (curcumin and hesperetin in a 1:1 mass ratio) using a mill (Tube Mill Control Mixer, IKA, Warsaw, Poland). The physical mixtures were then manually fed into the extruder’s hopper. The screw speed was set at 90 rpm, and the barrel temperature was adjusted to 135 °C, 150 °C, or 165 °C. After extrusion, the prepared extrudates were powdered using a mill (Tube Mill Control Mixer, IKA, Warsaw, Poland) and, subsequently, used for further study.

#### 2.2.3. Design of Experiment’s Settings

The Box–Behnken design was used as a tool to develop the statistical model and evaluate the significance of selected parameters, which were the contents of the active compounds, quantity of phosphatidylcholine in a carrier, and process temperature, on the solubility of curcumin and hesperetin. The analysis was performed using Statistica 13.3 (TIBCO Software Inc., Palo Alto, CA, USA). The significance level was *p* < 0.05. Based on Pareto plots and response surface plots, the statistically significant effects were determined. The experimental runs are shown in Table 1.

#### 2.2.4. X-Ray Powder Diffraction (XRPD)

The X-ray diffraction patterns of the samples were obtained using a Bruker AXS D2 Phaser diffractometer (Bruker, Germany) with a copper anode (Cu-Kα—1.54 Å, 30 kV, and 10 mA). The measurement settings were configured to a 5–45° 2-theta range, with a step size of 0.02° and a counting time of 2 s per step.

#### 2.2.5. Differential Scanning Calorimetry (DSC)

Thermal analysis was performed using a DSC 214 Polyma differential scanning calorimeter (Netzsch, Selb, Germany). Samples weighing approximately 5–10 mg were placed in crimped aluminum pans with a small hole in the lid. To remove water, the samples were heated to 80 °C for 8 min, cooled to 25 °C, and then heated again up to 280 °C. Raw compounds were heated to 280 °C, cooled to 25 °C, and heated again to 280 °C to determine their glass transition temperature (Tg). The experiments were conducted under a nitrogen atmosphere with a flow rate of 30 mL/min and heating and cooling rates of 10 °C/min. The Tg was defined as the midpoint between the onset and endpoint temperatures.

The Tg values of the PVP K30–phospholipid/PVP K30–xylitol mixtures were evaluated using a heating–cooling–heating cycle. The first heating step was performed to eliminate any remaining water. The sample was heated to 200 °C at 20 °C/min, cooled to 25 °C at 10 °C/min, and then reheated to 200 °C at 20 °C/min.

#### 2.2.6. Fourier-Transform Infrared Spectroscopy (FTIR-ATR)

FTIR-ATR spectra were recorded using a Shimadzu IRTracer-100 spectrometer (Shimadzu, Kyoto, Japan), equipped with a QATR-10 single bounce diamond extended range, and controlled by LabSolutions IR software (version 1.86 SP2, Warsaw, Poland), with a resolution of 1 cm^−1^. The compounds were prepared in their amorphous forms using DSC, as described in the differential scanning calorimetry section.

#### 2.2.7. High-Performance Liquid Chromatography (HPLC) Analysis

HPLC was employed to measure the concentrations of curcumin and hesperetin in samples collected during solubility, dissolution, and permeability studies. A Shimadzu LC-2050C system (Shimadzu Corp., Kyoto, Japan) equipped with a diode array detector (DAD) was used. The stationary phase consisted of a Dr. Maisch ReproSil-Pur Basic-C18 100 Å column with a 5 µm particle size and dimensions of 100 × 4.60 mm (Dr. Maisch, Ammerbuch-Entringen, Germany). The mobile phase was a mixture of HPLC-grade methanol and 0.1% acetic acid (80:20 *v*/*v*), filtered through a 0.45 µm nylon filter (Phenomenex, Torrance, CA, USA). The analysis was performed at a flow rate of 0.6 mL/min, with detection wavelengths of 420 nm for curcumin and 288 nm for hesperetin and a column temperature of 30 °C. The injection volumes were 2 µL for the solubility study and 10 µL for the dissolution and permeability tests. The run time was 7 min. The retention times for curcumin and hesperetin were 4.136 min and 2.651 min, respectively. The method’s validation parameters are provided in the Supplementary Materials (Table S1).

#### 2.2.8. Solubility Studies

An excess amount of powder (equivalent to 7.5 mg of each active substance) was placed in a 10 mL glass tube. Then, 2.0 mL of phosphate buffer (pH 6.8) was added, and the mixture was stirred at 100 rpm at room temperature (25 ± 0.1 °C) for 24 h. The resulting solutions were diluted 1:20 *v*/*v* with water, filtered through a 0.2 μm PTFE membrane filter (Sigma-Aldrich, St. Louis, MO, USA), and analyzed using the HPLC method described earlier. The analysis was conducted in triplicate. The phosphate buffer was prepared from concentrate following the manufacturer’s instructions (Stamar, Dąbrowa Górnicza, Poland).

#### 2.2.9. Dissolution Studies

The dissolution study was conducted using the paddle apparatus (Agilent 708-DS dissolution apparatus, Santa Clara, CA, USA). A 10 mg equivalent of extrudate for each active ingredient was added to vessels containing 250 mL of phosphate buffer at pH 6.8. The temperature was maintained at 37 ± 0.1 °C, and the paddle speed was set to 50 rpm. At the following time points: 5 min, 15 min, 30 min, 1 h, 2 h, 3 h, 4 h, 5 h, and 6 h, 2.0 mL of the sample was withdrawn and replaced with an equal volume of media equilibrated to the same temperature. The samples were then filtered through a 0.2 μm PTFE membrane filter, and HPLC analysis was conducted. Each sample was tested in triplicate.

#### 2.2.10. Permeability Studies

The Parallel Artificial Membrane Permeability Assay (PAMPA) models were employed to investigate the permeability of the blood–brain barrier (BBB) and the gastrointestinal tract (GIT) in vitro. The system consisted of two 96-well microfilter plates arranged in a sandwich configuration. The PAMPA system had two chambers, the acceptor chamber on top and the donor chamber on the bottom, separated by a 120 μm thick PVDF membrane coated with a 20% (*w*/*v*) dodecane solution of a lecithin mixture (Pion, Inc., Forest Row, East Sussex, UK). The acceptor and donor solutions were supplied by the manufacturer. Following the manufacturer’s instructions, the donor solution was diluted, and the pH was adjusted to approximately 6.8 for the GIT assay and 7.4 for the BBB assay using 0.5 M NaOH. After assembling the plates, both models were incubated for four hours at 37 °C in a humidity-saturated environment. The samples for the donor compartments were prepared as described in the solubility study. They were then diluted 1:5 (*v*/*v*) with water, filtered through a 0.2 μm PTFE membrane filter, and, subsequently, diluted 1:1 (*v*/*v*) with DMSO. The solutions were mixed with the donor solution for the GIT and BBB tests and filtered again. Finally, the resulting solutions were placed into the donor compartments. The compound’s concentration in the acceptor solution was used to express the results.

## 3. Results and Discussion

This study aimed to develop and characterize amorphous PVP K30–phosphatidylcholine dispersions of curcumin and hesperetin. The combination of amorphous polymeric dispersions and phospholipids represents a promising solubility-enhancing formulation. By leveraging the biopharmaceutical benefits of the amorphous form and the solubilizing capability of phospholipids, this approach is particularly valuable for poorly soluble compounds, as low solubility limits their therapeutic potential. By increasing the solubility, amorphous solid dispersions can enhance drug permeability by stimulating passive diffusion, raising the concentration of free molecules capable of crossing biological membranes, such as enterocyte membranes [37]. Additionally, it has been shown that amorphous dispersions offer advantages over other well-established methods for improving solubility [38]. In a study on the biopharmaceutical enhancement of etoposide, amorphous dispersion was the only method that not only increased solubility but also enhanced permeability. In contrast, the use of cyclodextrins, solubilizers, and cosolvents increased solubility but resulted in decreased permeability [38]. This research highlights the amorphous dispersion approach as a promising bioavailability-enhancing solution.

On the other hand, the use of phospholipids is a well-recognized technique for formulating drug delivery systems with improved bioavailability. In a study on mangiferin, a polyphenol abundantly found in *Mangifera indica*, the authors fabricated a phospholipid complex that enhanced intestinal permeability, leading to a higher plasma concentration in rats after oral administration [39]. Interestingly, research has shown that the curcumin–phospholipid combination is an effective way to increase curcumin’s bioavailability. By forming curcumin–phospholipid complexes, the solubility of curcumin was improved, resulting in better bioavailability and enhanced hepatoprotective activity in a rat model [40]. Another study highlighting the beneficial effect of phospholipids on the bioavailability of plant compounds was reported by Saoji et al. In their research, the authors obtained complexes of standardized Bacopa extract with phospholipids, which exhibited improved water solubility and a better dissolution profile. These effects were attributed to partial amorphization and the amphiphilic nature of the phospholipid, which facilitated the solubilization of the active compounds [41].

When developing phospholipid systems, one may encounter technological challenges, as there is a tendency to form lipidic complexes that are unsuitable for solid dosage forms. However, the addition of an excipient to the final formulation can help address this issue. For example, Neusilin US was used to obtain powdered phosphatidylcholine–celecoxib dispersions [42]. In another study, trehalose was employed to solidify the phospholipid powder [43]. Additionally, one common issue when working with solid phospholipid complexes is the aggregation and agglomeration phenomenon, which negatively impacts the dissolution and absorption of active pharmaceutical ingredients [44].

Other researchers have explored the fabrication of polymer–phospholipid dispersions. For example, PVP K30 was combined with a phospholipid to create a baicalein complex. Notably, the baicalein–phospholipid–polymer system not only demonstrated higher baicalein dissolution but also improved bioavailability after oral administration in a rat model compared to the baicalein–phospholipid complex. The authors speculated that these results were due to several factors, including the amorphous form of the active compound, the permeability-boosting properties of the phospholipid, the preservation of the baicalein–phospholipid complex, and the enhanced dispersibility of the system provided by the PVP K30 matrix [44]. Similarly, Lale et al. demonstrated the superiority of ternary drug–polymer–phospholipid dispersions [45]. In their solubility study, the aprepitant–polymer–phospholipid system showed a greater improvement in solubility compared to the aprepitant-phospholipid system. The authors attributed these findings not only to the solubilizing activity of the phospholipid but also to the potential formation of colloidal drug–polymer structures or nanosized drug-rich submicron particles (nanodroplets), which likely acted as reservoirs, releasing the drug into the medium. These additional solubility-enhancing mechanisms resulted from the polymer’s inclusion in the dispersion. Furthermore, ternary systems performed better in an in vivo bioavailability study in a rat model, yielding higher C_max_ and AUC values compared to drug–phospholipid systems [45]. These studies demonstrate the advantage of active-polymer–phospholipid formulations over simple active-phospholipid complexes. The use of polymer not only improves the texture for solid form production but also offers a clear benefit over basic phospholipid systems, with the added potential for using hot-melt extrusion to produce systems incorporating phospholipids.

This research emphasizes the use of phosphatidylcholine from egg yolk. Egg yolk phosphatidylcholine is reported to contain a higher concentration of phosphatidylcholine than soybean phosphatidylcholine [46]. Therefore, one can expect better solubilizing capacity due to the presence of phosphatidylcholine. Additionally, there are reports on the benefits of using egg yolk phosphatidylcholine in treating neurological disorders such as Alzheimer’s disease. Given that the studied polyphenol combination is expected to act in neurodegenerative diseases, this mode of action is advantageous [46].

**Figure 1:**
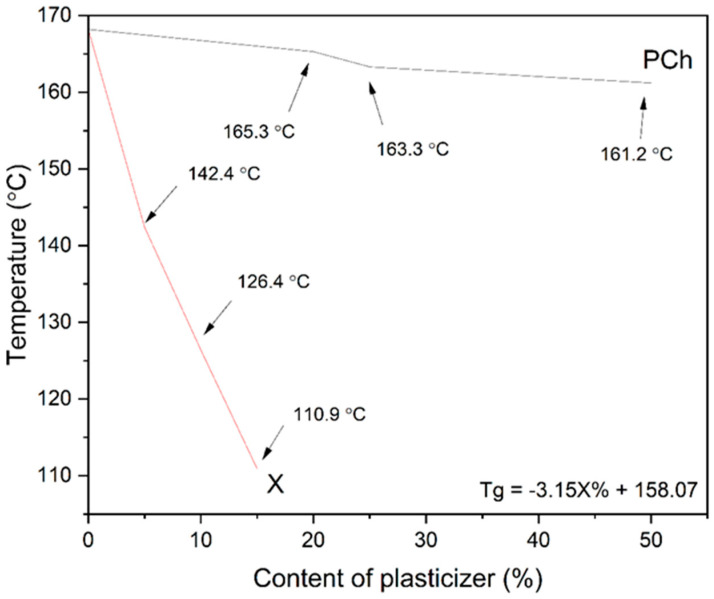
The dependence of the xylitol (X) and phosphatidylcholine (PCh) contents on the Tg value of the PVP K30–excipient blend. Arrows point to the Tg. X stands for xylitol, while PCh stands for phosphatidylcholine. The equation describes the impact of the xylitol percentage on PVP K30 glass-transition temperature.

To fabricate amorphous PVP K30–phospholipid dispersions using hot-melt extrusion, we first aimed to design a PVP K30–phospholipid blend with thermal properties suitable for the process. Initially, we evaluated whether phosphatidylcholine exhibits a plasticizing effect on PVP K30. Our results show that phosphatidylcholine has a minimal plasticizing effect on the polymer (Figure 1). Therefore, additional substances with significant plasticizing effects were needed. We chose xylitol, a common sweetener known for its plasticizing effect on PVP K30 [47], which was confirmed by our analysis (Figure 1). The data show a linear relationship between xylitol contents (5–15%), allowing us to determine the equation of the linear function and accurately calculate the required amount of xylitol to achieve the targeted glass transition temperature (Tg) for the PVP K30–phosphatidylcholine–xylitol blend. For efficient hot-melt extrusion, the Tg of the extruded mixture must be at least 20 °C higher than the process temperature [48,49]. Accordingly, the carriers were designed to have a Tg of approximately 115 °C. The choice of glass transition temperature was determined by the need to achieve a compromise between smooth processing and stability, which results from the inhibition of molecular mobility provided by the carrier. To obtain the blend with the desired properties, 13.67% xylitol was added to PVP K30, while blends containing 20% and 40% phosphatidylcholine required 13.04% and 11.77% xylitol, respectively. Before using hot-melt extrusion, it is necessary to determine whether the active substance degrades at the planned processing temperature. It has been indicated that curcumin starts to degrade at 245 °C [50], while hesperetin degrades at 205 °C [51]. The temperatures used in our hot-melt extrusion processing are below these degradation temperatures.

After determining the composition of the carrier, we focused on evaluating whether the selected formulation and process factors significantly affect the solubility of the active compounds. To achieve this, we employed the Design of Experiment (DoE) approach, which allowed us to systematically prepare the formulations. We used the Box–Behnken design, a widely used method in pharmaceutical formulation optimization [52,53]. As independent factors (inputs), we selected the following: (i) the amount of active compounds in the formulation (curcumin:hesperetin mass ratio of 1:1), (ii) the amount of phosphatidylcholine in the carrier, and (iii) the temperature at which the hot-melt extrusion was performed. Solubility of curcumin and hesperetin was chosen as the dependent factor (output).

**Figure 2:**
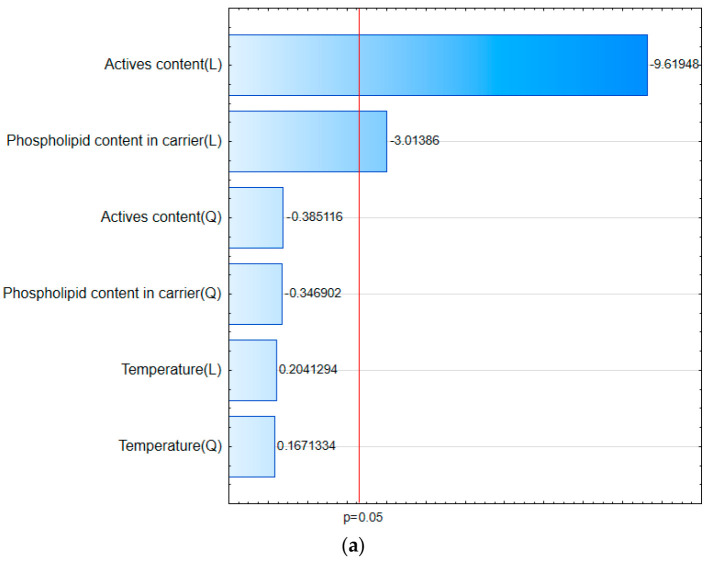

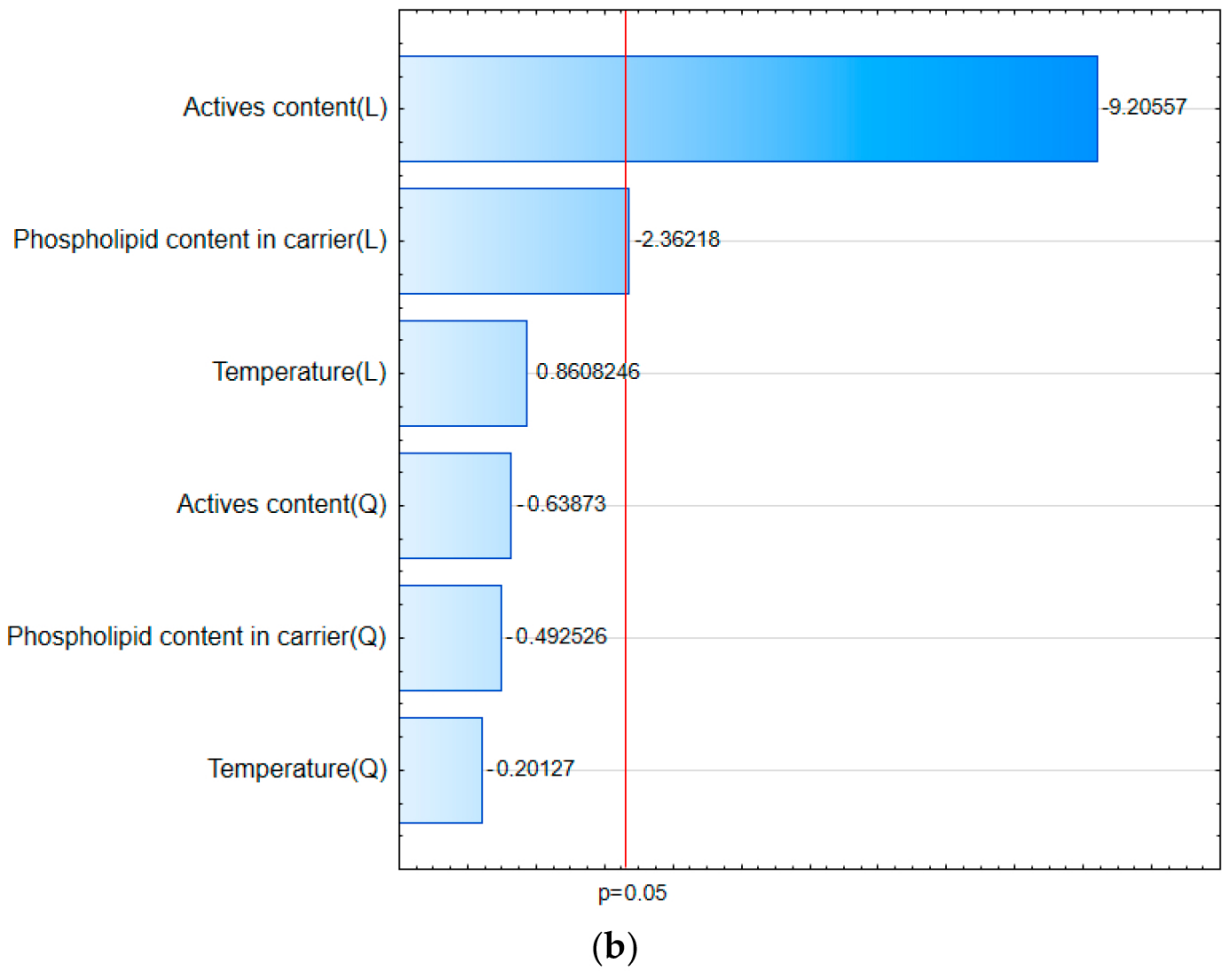
Pareto chart of the solubility of (**a**) curcumin and (**b**) hesperetin. Red line indicates statistical significance.

Adopting a linear model for both curcumin and hesperetin, Pareto charts (Figure 2a,b) reveal that the statistically significant factors affecting the solubility of the active compounds are the amount of active compounds and the amount of phosphatidylcholine in the carrier. These factors have a negative effect, meaning that lower content of active compounds or phosphatidylcholine results in a greater increase in solubility. The process temperature, however, was found to have no significant impact on the solubility of the compounds.

**Figure 3:**
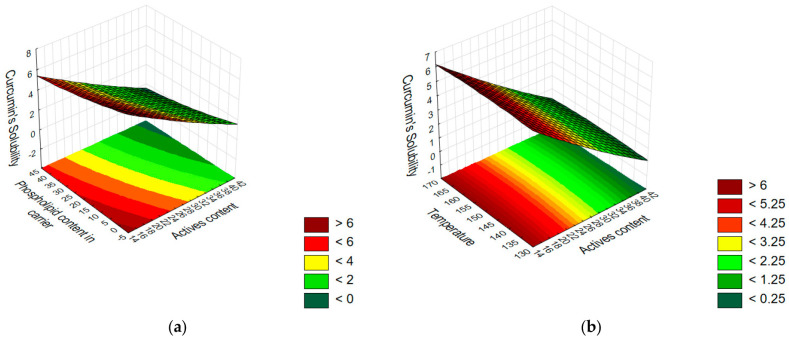

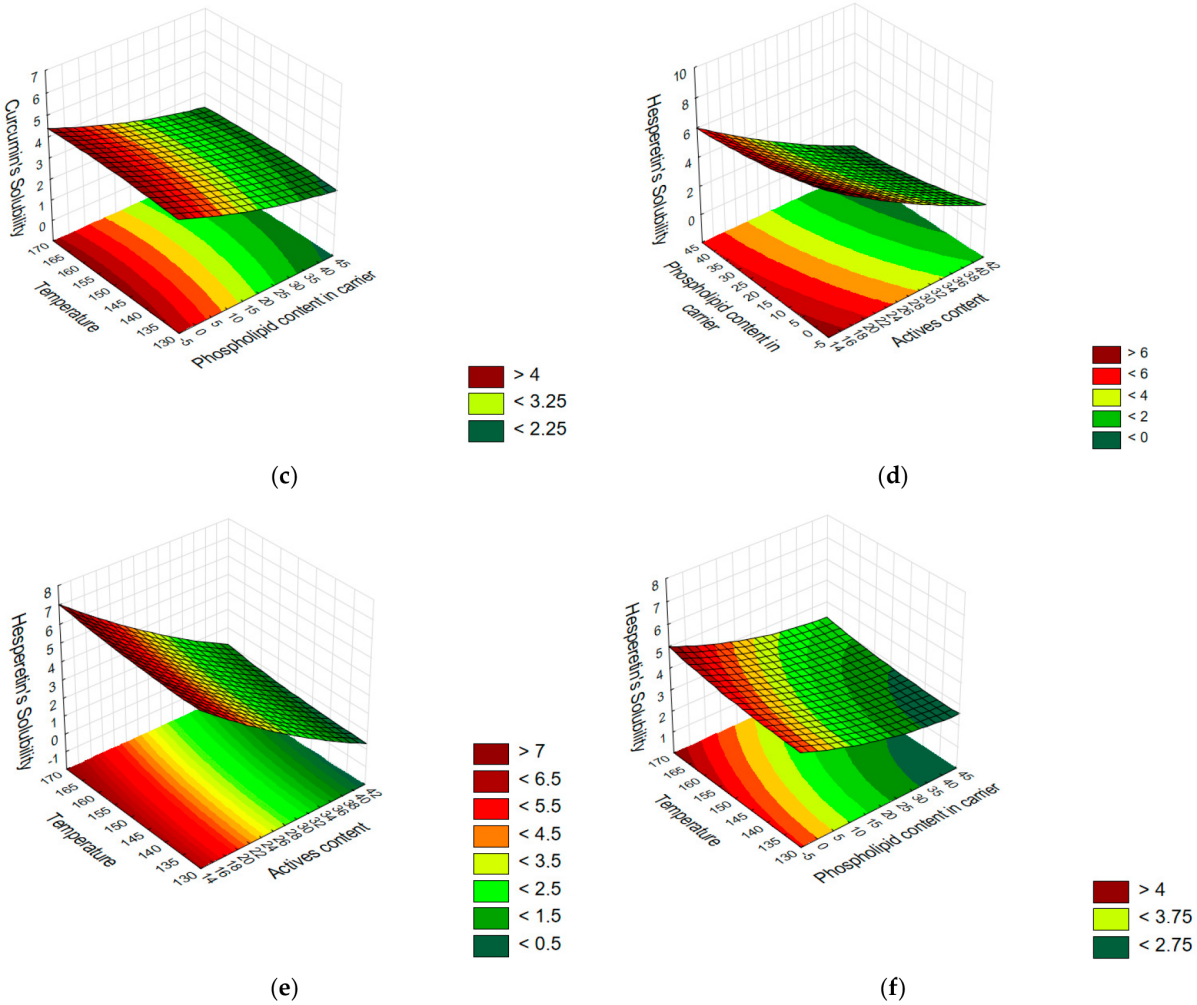
Response surface plots presenting the effect of the active and phospholipid contents (**a**,**d**); active content and process temperature (**b**,**e**); and phospholipid content and process temperature (**c**,**f**) on the solubility of curcumin (**a**–**c**) and hesperetin (**d**–**f**).

Furthermore, the analysis of the response surface (Figure 3), which graphically represents the relationship between various factors and their effect on solubility, supported the results of the model. It is evident that temperature does not influence the solubility of the active compounds. However, for both the amount of active compounds and the amount of phosphatidylcholine in the carrier, the response surface indicates that solubility increases as their contents in the formulation decreases. These observations align with the Pareto chart analysis. The obtained R^2^ and Radj values for the proposed models are high, demonstrating the good fit and applicability of the Box–Behnken design. Specifically, for the curcumin solubility model, R^2^ = 0.927 and Radj = 0.873, while for the hesperetin solubility model, R^2^ = 0.920 and Radj = 0.860.

The obtained models can be described by the following equations:

Curcumin’s solubility = 3.38557 − 0.267045 * X1 + 0.000999733 * X1 * X1 − 0.0555958 * X2 + 0.000351771 * X2 * X2 + 0.0941389 * X3 − 0.000301296 * X3 * X3(1)

Hesperetin’s solubility = 17.7187 − 0.314951 * X1 + 0.00177547 * X1 * X1 − 0.0562417 * X2 + 0.000534792 * X2 * X2 − 0.0996222 * X3 + 0.000388519 * X3 * X3(2)

where X1 is the content of actives, X2 is phosphatidylcholine content, and X3 is processing temperature.

The factors mentioned above may significantly impact the performance of the formulation. The content of active ingredients affects their biopharmaceutical properties. It is indicated that a higher drug load leads to a decrease in the dissolution rate and the solubility achieved. On the other hand, a lower drug load favors polymer-controlled release, resulting in the rapid and complete release of the drug from the matrix [54,55]. The amount of phospholipid in the system is important for its solubilizing properties and for achieving the critical micelle concentration, which is the concentration above which micelle formation is observed. It is implied that there is an optimal drug–phospholipid ratio to ensure an increase in solubility, as demonstrated in a study by Fong et al., which explored the effect of the phospholipid content on the celecoxib solubility. The authors reported that the concentration of celecoxib increased with the amount of phospholipid in the formulation until a ratio of 50:1 (phospholipid: celecoxib) was reached; above this ratio, the concentration of the active compound began to decrease [56]. The process temperature is an important parameter when developing the hot-melt extrusion process. It affects the extrudability of the compound by causing the polymer to melt, providing the energy to break the crystal lattice [57]. An inadequate temperature can result in incomplete amorphization, leading to a partially crystalline extrudate [58].

The purpose of this research was to produce an amorphous PVP K30–phosphatidylcholine solubility-enabling formulation. Therefore, solubility determination was performed as part of the DoE project. In the solubility study, we observed a significant improvement in this property, as shown in Table 2.

Analyzing the results of the solubility study, it is evident that formulation F7 provided the highest solubility improvement, achieving concentrations of 6.648 mg/mL and 7.397 mg/mL for curcumin and hesperetin, respectively. This translates to an enhancement of 47,486-fold and 1479-fold. This system contained 15% active compounds and 20% phosphatidylcholine in the carrier and was produced at 165 °C. Interestingly, when examining the results for systems with 40% active compounds, the best formulation was F2, which used only a PVP K30–plasticizer blend as the carrier. In this case, the presence of phosphatidylcholine did not translate into a more advantageous effect on solubility, even though the phospholipid can directly interact with and entrap the dissolved molecule. This observation was also confirmed for systems containing 27.5% active compounds, where systems without phospholipids performed better. On the other hand, for formulations with 15% active compounds, system F1, the only dispersion based on a PVP K30–xylitol blend, exhibited worse solubility than other systems containing phospholipids (F3, F5, F7) at the same active compound percentage. We speculate that the ratio of active compounds to phospholipids may be a crucial factor. Only when there is a sufficiently large amount of phosphatidylcholine in the system does it show a solubility-enhancing effect. With a lower amount of phospholipid per molecule of the compound, the PVP K30 likely plays a more significant role as a stabilizer of the amorphous form, or as an agent that shifts the amorphous solubility limit, since the phospholipid cannot effectively demonstrate its solubilizing effect. As indicated by the DoE analysis, the amount of active compound is crucial for the performance of formulations in biopharmaceutical studies, as PVP K30 and phospholipid act more effectively as stabilizers of the amorphous form. Interestingly, the F6 system, which did not achieve full amorphization, was not characterized by the worst solubility. The poorest solubility improvement was reported for system F8 (40% active compounds, 20% phosphatidylcholine, and a process temperature of 165 °C).

**Figure 4:**
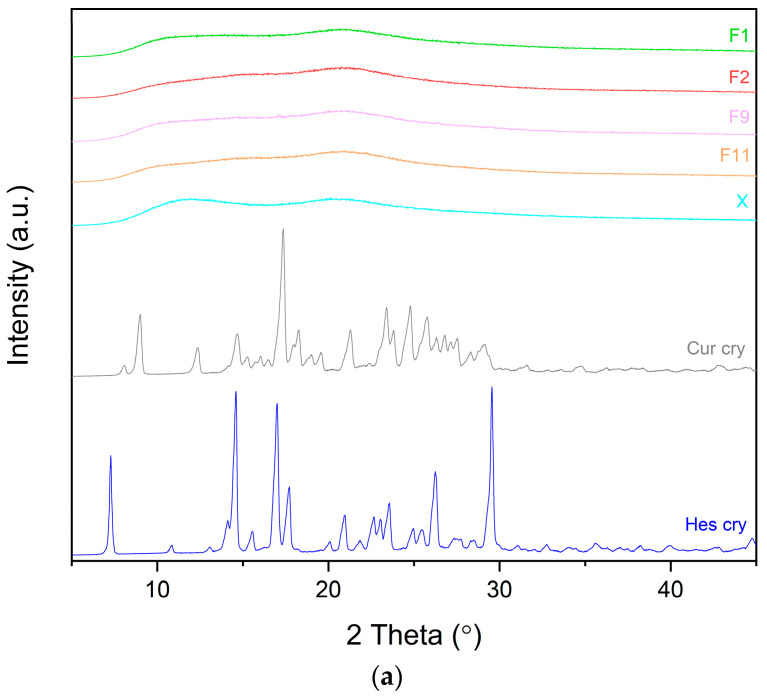

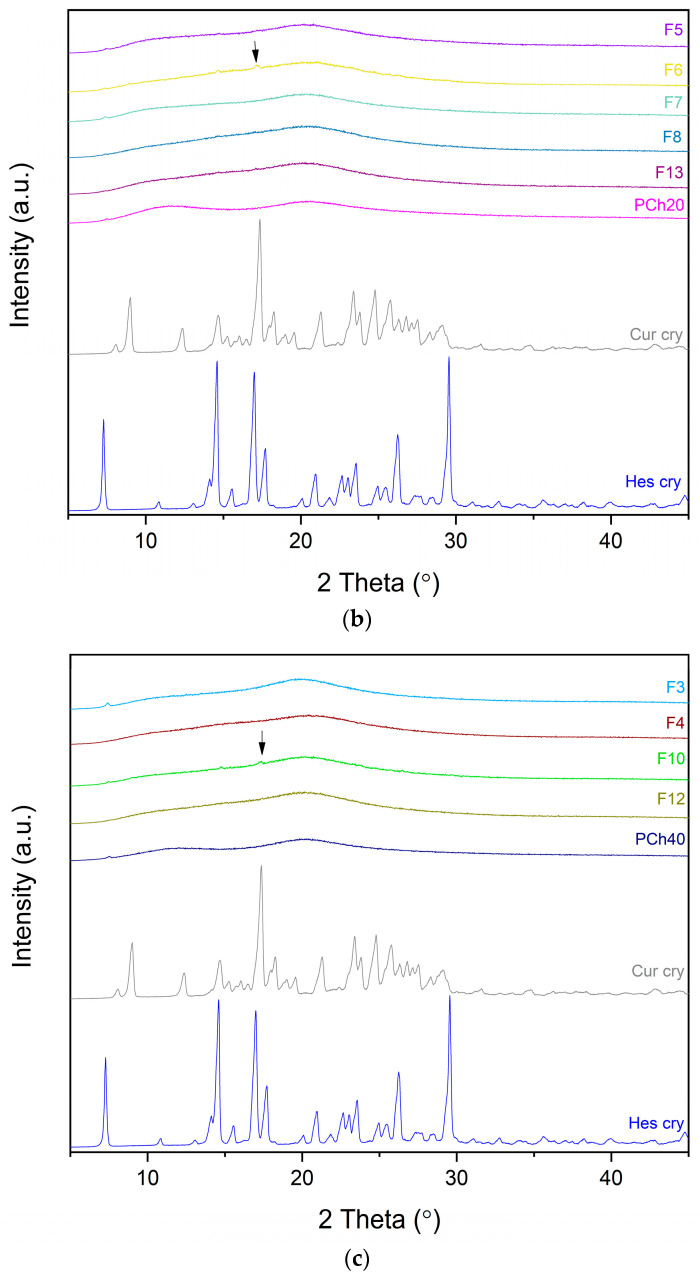
XRPD analysis of curcumin, hesperetin, formulations, and carriers: (**a**) formulations based on only the PVP K30–xylitol carrier; (**b**) formulations with a 20% content of phosphatidylcholine; (**c**) formulations with a 40% content of phosphatidylcholine. The arrow points peak.

After performing the DoE analysis, we proceeded with an investigation into the characteristics of the produced formulations. The solid-state identification began with X-ray powder diffraction (XRPD). In this analysis, we aimed to determine whether the hot-melt extrusion processing led to the production of amorphous dispersions. The diffractograms of the extruded systems of curcumin and hesperetin are presented in Figure 4. The raw compounds exhibited sharp peaks as follows: curcumin at 8.85°, 17.24°, 21.16°, 23.27°, 24.67°, 24.68°, and 25.63° 2theta and hesperetin at 7.33°, 14.64°, 17.10°, 17.82°, 21.08°, 26.39°, and 29.63° 2theta. Upon examining the XRPD diffractograms of the formulations, we observed a significant reduction in crystallinity, indicating an amorphous character due to the disappearance of sharp Bragg peaks. However, small peaks in formulations F6 and F10 were still noticeable, suggesting incomplete amorphization in some of the tested samples. It is difficult to explain this observation. Both systems were obtained at 135 °C, and we speculate that the process temperature of 135 °C was not high enough to achieve molecular miscibility of the components. However, formulations prepared at the same temperature (F5, F9) were fully amorphous. This suggests that a combination of factors, such as the active content and process temperature, might have influenced residual crystallinity. The 135 °C temperature may not have provided sufficient energy to disrupt the crystal lattice during hot-melt extrusion of the actives-rich blend in those systems. It is worth noting that curcumin and hesperetin have good glass-forming abilities, so it is unlikely that the compounds crystallized after extrusion. Based on these findings, we can conclude that we successfully created amorphous PVP K30–phosphatidylcholine dispersions comprising curcumin and hesperetin through hot-melt extrusion.

**Figure 5:**
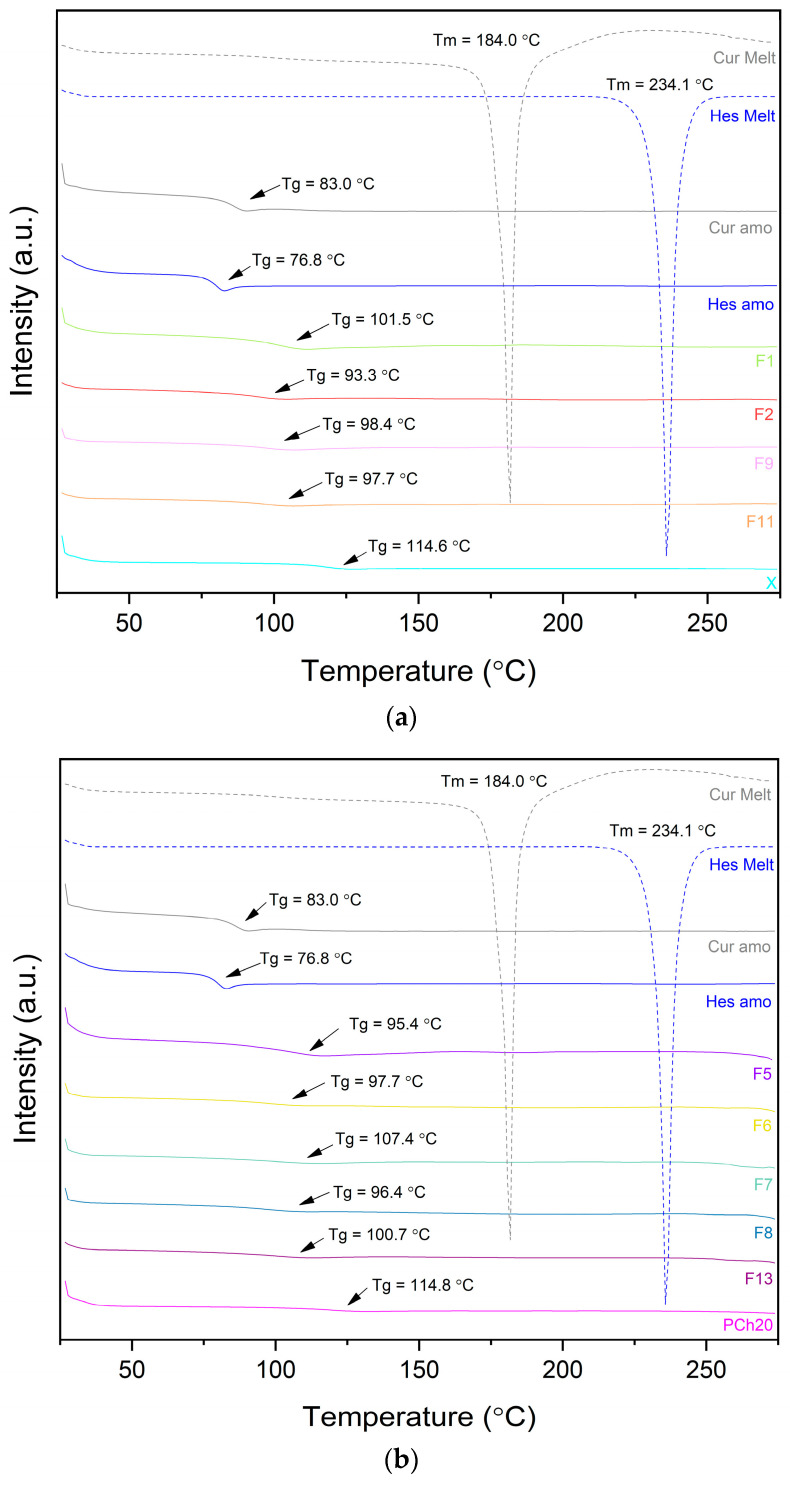

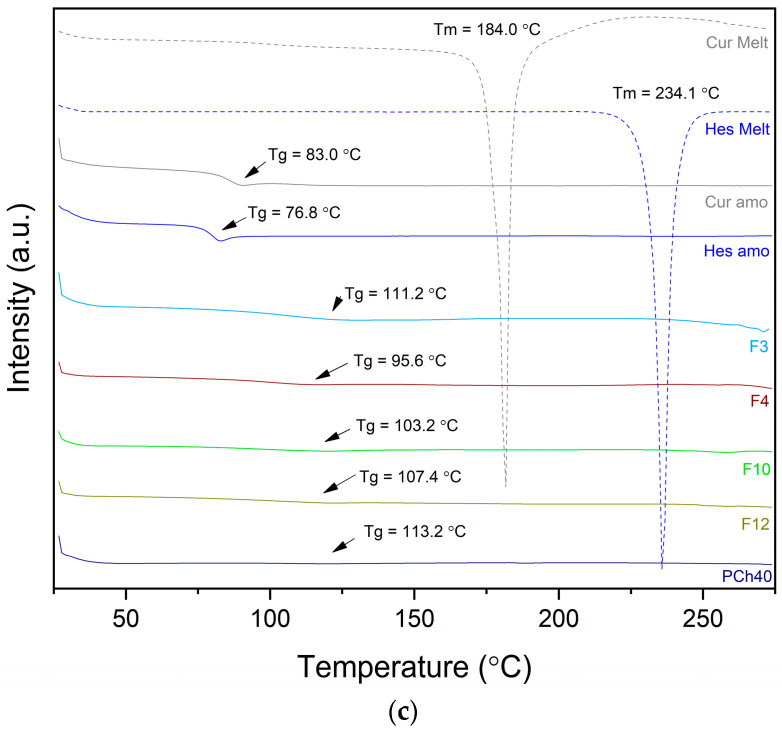
DSC analysis of curcumin, hesperetin, formulations, and carriers (**a**) formulations based on only PVP K30–xylitol carrier, (**b**) formulations with a 20% content of phosphatidylcholine, (**c**) formulations with a 40% content of phosphatidylcholine. Arrows point at Tg values.

The thermal properties of the fabricated formulations were investigated using Differential Scanning Calorimetry (DSC). The DSC thermograms (Figure 5) of the prepared dispersions show a single glass transition temperature (Tg), indicating good miscibility of the components and suggesting that the active compounds are molecularly dispersed within the carriers [59,60]. Moreover, no endothermic peaks corresponding to the melting points of the active compounds were detected. This observation contrasts with the findings from the XRPD analysis, which suggested the presence of crystalline traces. It is possible that the residual crystals dissolved into the dispersion matrix during the heating process. The Tg values of the formulations fall between the Tg values of the active compounds (curcumin Tg = 83.0 °C and hesperetin Tg = 76.8 °C) and the carriers (X Tg = 114.6 °C, PCh20 Tg = 114.8 °C, and PCh40 Tg = 113.2 °C). In this case, the active compounds demonstrate a Tg-reducing effect, acting as plasticizers, while the carriers, with higher Tg values, impart an anti-plasticizing effect on the active compounds [61,62]. Consequently, the carriers decrease the molecular mobility of the active molecules, ensuring the stability of the amorphous form, as predicted by the Gordon–Taylor equation. Based on the percentage of components, mixing compatible materials with different Tg values should result in a single-phase system, with a Tg temperature within the range of the Tg values of the individual components, assuming their miscibility [63]. This principle applies to the PVP K30–phosphatidylcholine dispersions developed in our study.

**Figure 6:**
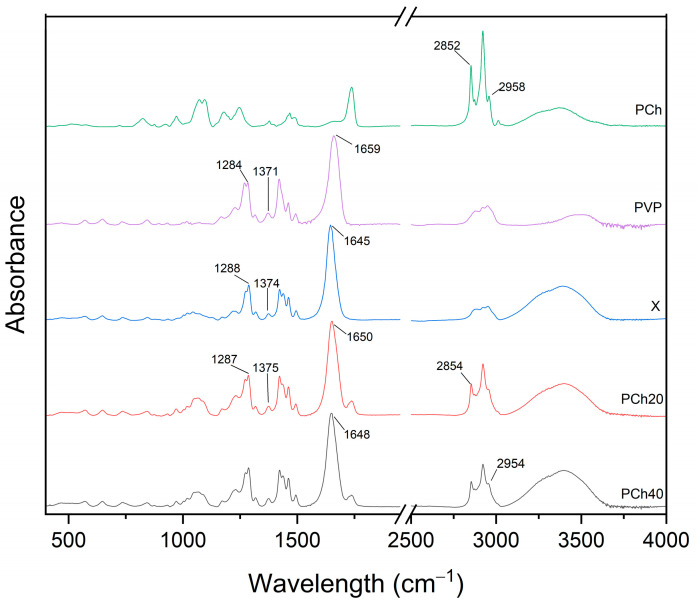
FTIR-ATR spectra of PVP K30, phosphatidylcholine (PCh), and the modified polymers (PVP K30–xylitol (X), PVP K30–xylitol–20% phosphatidylcholine (PCh20), and PVP K30–xylitol–40% phosphatidylcholine (PCh40) blends).

To investigate potential molecular interactions in the carrier blends and formulations, we performed an FT-IR/ATR (Fourier-Transform Infrared Spectroscopy) analysis. Comparing the spectra of the modified carriers (Figure 6) with those of phosphatidylcholine and PVP K30 revealed some shifts in the peaks, indicating interactions among the components at the molecular level. The peaks at 2958 cm^−1^ and 2852 cm^−1^ of phosphatidylcholine shifted to 2954 cm^−1^ and 2854 cm^−1^, respectively, in both phosphatidylcholine-containing carriers. Conversely, the peak at 1659 cm^−1^, characteristic of PVP K30, shifted to 1645 cm^−1^, 1650 cm^−1^, and 1648 cm^−1^ for X, PCh20, and PCh40, respectively. Additionally, the 1371 cm^−1^ peak from PVP K30 in the X spectrum is seen at 1374 cm^−1^, while for the phosphatidylcholine carriers, it shifted to 1375 cm^−1^. Finally, the peaks at 1288 cm^−1^ in the X spectrum and at 1287 cm^−1^ in the phosphatidylcholine-containing carriers correspond to the 1284 cm^−1^ peak of PVP K30.

**Figure 7:**
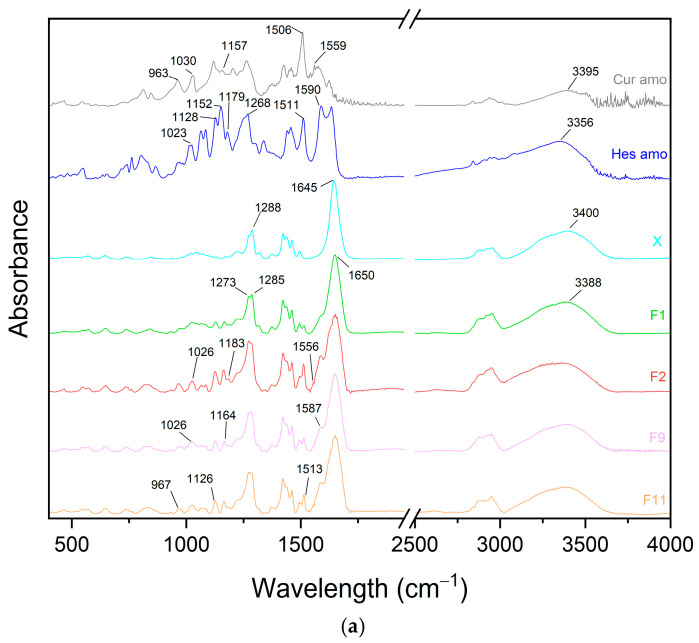

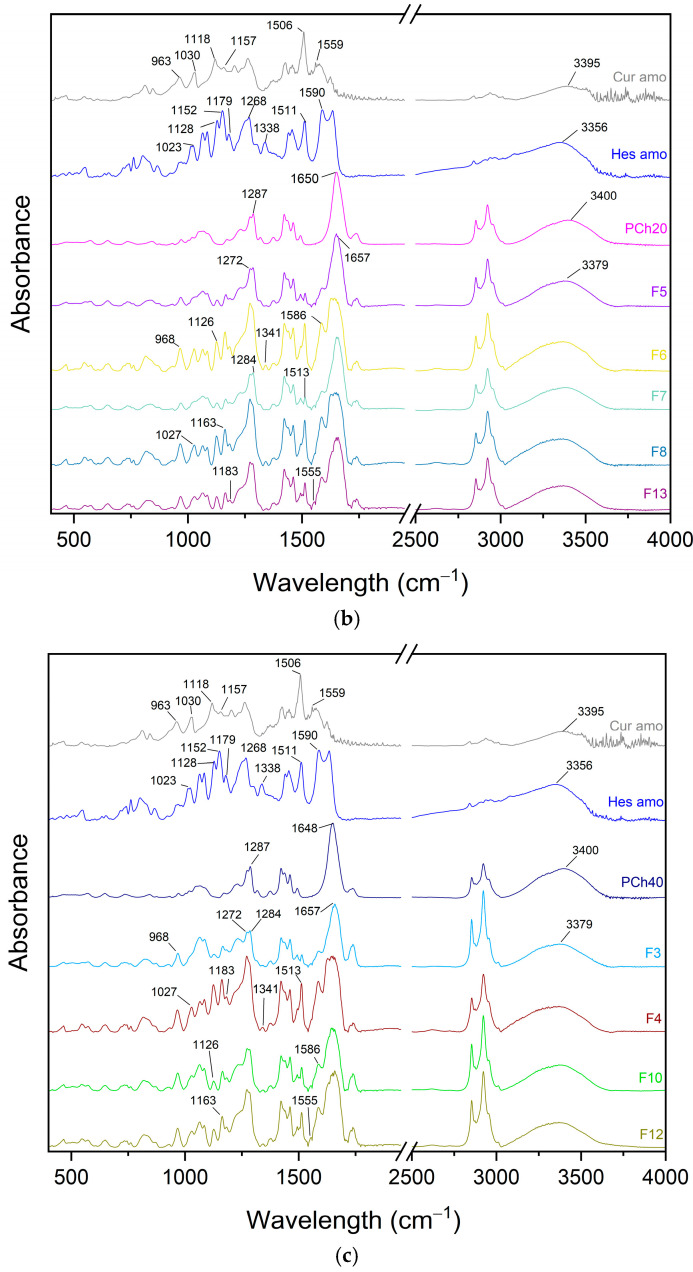
FTIR-ATR spectra of the amorphous compounds, formulations, and carriers. They are grouped in terms of the used carrier: (**a**) PVP K30–xylitol (X); (**b**) PVP K30–xylitol–20% phosphatidylcholine (PCh20); (**c**) PVP K30–xylitol–40% phosphatidylcholine (PCh40).

The spectra of the formulations were grouped according to the carrier used and compared with the spectra of the active compounds in their amorphous form and the carriers (Figure 7). In these spectra, peak shifts were observed, suggesting the formation of interactions among the components of the dispersion. For systems composed of PVP K30 and plasticizer as the carrier, a broad peak at 3388 cm^−1^ was noted, which can be attributed to peaks at 3356 cm^−1^ (hesperetin), 3395 cm^−1^ (curcumin), and 3400 cm^−1^ (carrier). Additionally, the peak at 1645 cm^−1^ of the carrier shifted to 1650 cm^−1^ in the formulations. The peak at 1587 cm^−1^ in the systems can be assigned to the 1590 cm^−1^ peak present in the spectrum of hesperetin. The peak of curcumin at 1559 cm^−1^ shifted to 1556 cm^−1^ in the dispersions. The peak at 1513 cm^−1^ in the formulations is likely associated with the peak of curcumin at 1506 cm^−1^ or the slightly shifted peak of hesperetin at 1511 cm^−1^. Another set of peaks indicating interactions is seen at 1285 cm^−1^ and 1273 cm^−1^, which correspond to the peak at 1288 cm^−1^ of the carrier and the peak at 1268 cm^−1^ of hesperetin. Further shifts were observed, with the peak at 1179 cm^−1^ of hesperetin moving to 1183 cm^−1^ in the formulations. The peak at 1164 cm^−1^ in the dispersions is likely due to the 1152 cm^−1^ peak of hesperetin or the 1157 cm^−1^ peak of curcumin. Similarly, the peak at 1126 cm^−1^ corresponds to 1128 cm^−1^ of hesperetin or 1118 cm^−1^ of curcumin, while the peak at 1026 cm^−1^ in the dispersion is probably a shifted version of the 1030 cm^−1^ peak of curcumin or the 1023 cm^−1^ peak of hesperetin. Lastly, the peak of curcumin at 963 cm^−1^ shifted to 967 cm^−1^ in the formulation spectra.

Further analysis of the spectra from systems where the carrier was composed of PVP K30, plasticizer, and phosphatidylcholine revealed the presence of a peak at 3379 cm^−1^, which can be attributed to peaks at 3356 cm^−1^ (hesperetin), 3395 cm^−1^ (curcumin), and 3400 cm^−1^ (carrier). The peak at 1657 cm^−1^ in the formulation corresponds to the peaks at 1650 cm^−1^ for PCh20 and 1648 cm^−1^ for PCh40. The peak at 1590 cm^−1^ from hesperetin shifted to 1586 cm^−1^ in the dispersion, while the curcumin signal at 1559 cm^−1^ shifted to 1555 cm^−1^ in the systems. The peak at 1513 cm^−1^ in the systems can be attributed to the peak at 1506 cm^−1^ of curcumin or 1511 cm^−1^ of hesperetin. Additionally, the peak at 1341 cm^−1^ corresponds to the peak at 1338 cm^−1^ of hesperetin. The peak of the carrier at 1287 cm^−1^ shifted to 1284 cm^−1^, while the peak of hesperetin at 1268 cm^−1^ shifted to 1272 cm^−1^ in the formulations. The dispersion peak at 1183 cm^−1^ corresponds to the 1179 cm^−1^ peak of hesperetin, and the peak at 1163 cm^−1^ is likely associated with the 1152 cm^−1^ peak of hesperetin or the 1157 cm^−1^ peak of curcumin. The signal at 1126 cm^−1^ in the system corresponds to the shifted peak of 1128 cm^−1^ from hesperetin or 1119 cm^−1^ from curcumin. Finally, the peak at 1027 cm^−1^ in the dispersions may correspond to the 1030 cm^−1^ peak of curcumin or the 1023 cm^−1^ peak of hesperetin. A shift in the 963 cm^−1^ peak of curcumin to 968 cm^−1^ in the systems is also evident.

**Figure 8:**
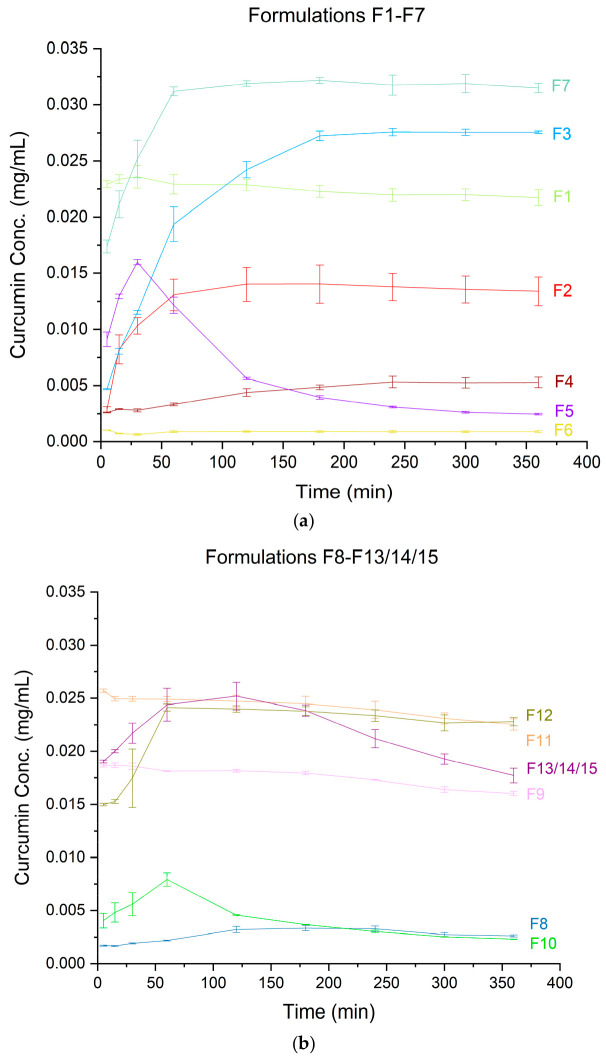

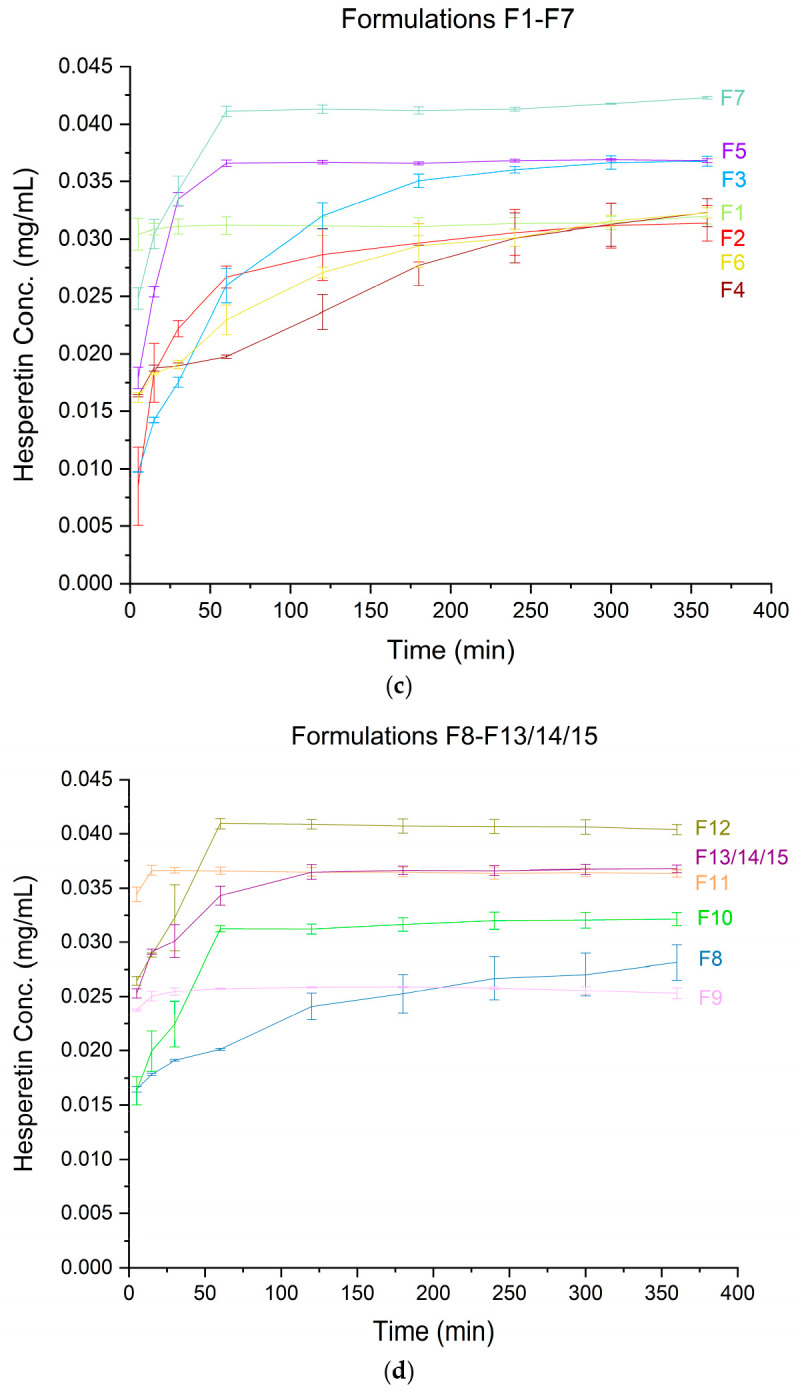
Dissolution rate profiles for the amorphous systems of curcumin (**a**) F1–F7 and (**b**) F8–F13/14/15 and hesperetin (**c**) F1–F7 and (**d**) F8–F13/14/15.

Systems based on the same carrier exhibited the same shifts in the spectra, although the intensity of individual peaks varied depending on the amounts of the active ingredients in the formulation. Continuing with our investigation, we conducted dissolution studies to examine the release kinetics (Figure 8) and verify whether the supersaturated state would be maintained.

A dissolution profile provides insight into the biopharmaceutical performance of a formulation. Analyzing the different profiles for curcumin, it is evident that the formulations based on PVP K30 alone, such as F1, F9, and F11, exhibited very rapid dissolution, reaching a plateau at the first measurement point (5 min). However, the F2 profile, which also only uses PVP K30 as the carrier, achieves a plateau after approximately 1 h. The difference among these formulations (F1, F2, F9, and F11) lies in the content of the active compound, with F2 containing 40% active compounds. This suggests that a higher drug load can overcome the beneficial effect of PVP K30 on wettability. Formulations containing phosphatidylcholine show a more gradual dissolution of the active compounds, reaching a plateau at different times depending on the system. For instance, F7 (20% phosphatidylcholine) reaches a plateau after about 1 h, while F3 (40% phosphatidylcholine) takes around 3 h. This indicates that higher phospholipid content slows the dissolution. The F5, F10, and F13/14/15 profiles exhibited a poor “parachute” phenomenon, where the concentration of active compounds decreases after reaching a maximum concentration. This effect was the most delayed in the F13/14/15 profile For hesperetin, the F1, F9, and F11 systems also showed near-immediate and complete dissolution. Other profiles showed a gradual release until reaching a plateau. Interestingly, F4, F6, and F8 exhibited a continuous dissolution over the 6 h duration of the experiment. Notably, curcumin and hesperetin displayed different dissolution profiles, likely due to their distinct crystallization tendencies. Some curcumin profiles showed a decrease in the concentration, suggesting crystallization, while the hesperetin profiles did not exhibit this phenomenon.

The DoE model highlighted the statistical significance of the phospholipids as a factor influencing the solubility, indicating that lower amounts of phospholipids lead to greater improvement in the solubility. However, the F7 formulation, which contains 20% phosphatidylcholine, exhibits the best biopharmaceutical properties. This suggests that there is an optimal phospholipid content, where a modest addition enhances the solubility.

The spring and parachute effect, characteristic of supersaturated formulations, is clearly evident in the release profiles. This phenomenon refers to fluctuations in the concentration of the dissolved compound over time. In the initial phase of dissolution, a supersaturated state is achieved in the aqueous solution due to the presence of the compound in its amorphous form [28]. During the second phase, stabilization of this supersaturation is expected to ensure a prolonged absorption window from the gastrointestinal tract [64,65]. In the case of amorphous polymer–phospholipid dispersions, both excipients played a role in stabilizing the amorphous form in the solution, namely, the polymer and the phospholipid. We hypothesize that a synergistic effect between the phospholipid and PVP K30 contributed to improving the solubility and the maintenance of the supersaturated state. Phosphatidylcholine facilitated the formation of micellar structures that solubilized the active compounds while simultaneously preventing crystal nucleation through direct encapsulation of the molecules, thus stabilizing the supersaturated state. On the other hand, the polymer helped prolong the supersaturation by hindering nucleation, inhibiting crystal growth, and possibly forming colloidal drug-rich particles.

It is crucial to understand the self-organizing behavior of phospholipids in an aqueous environment, including within the intestine, as it significantly influences their potential to enhance the bioavailability of compounds. Phospholipids possess amphiphilic properties, which enable them to spontaneously organize in water. When a poorly soluble compound is present, the phospholipid can encapsulate the molecule within a micelle, thereby solubilizing it [66,67]. This solubilizing ability allows for phospholipids to stabilize the supersaturated state, as demonstrated by Fong et al. [56]. In their study, the authors created a solid dispersion of celecoxib with phospholipids. The resulting systems exhibited improved solubility compared to both the crystalline and amorphous forms of the compound. The study also highlighted that phospholipids not only enhanced solubility but also improved permeability, suggesting that the stabilized supersaturated state contributed to the increased membrane permeability [56].

Another important factor, alongside phosphatidylcholine, contributing to the prolongation of supersaturation is PVP K30. The stabilizing effect of polymeric excipients is well-documented, involving various mechanisms that hinder nucleation and crystal growth. One such mechanism is the increase in the viscosity of the medium, which reduces the mobility of molecules [68]. Additionally, molecular interactions have been shown to alter molecular mobility, thereby enhancing the stability of the amorphous form and delaying the onset of crystallization [69]. The role of PVP K30 in maintaining the supersaturation state of curcumin is highlighted in a study by Nogami et al. The authors demonstrated that favorable intermolecular interactions between the pyrrolidone skeleton of PVP K30 and the curcumin molecule led to the formation of hydrogen bonds, contributing to the high apparent solubility of curcumin compared to the other polymers tested [70]. Furthermore, the adsorption of PVP K30 onto the crystal surface can impede crystal growth [58]. Additionally, the presence of a polymer may facilitate the formation of drug-rich nanoparticles. When the amorphous solubility is exceeded, phase separation occurs, resulting in the formation of a drug-rich colloidal phase stabilized by the polymer, and a water-rich phase. These phases exist in a metastable equilibrium, where the drug-rich phase acts as a reservoir, continuously releasing the active ingredient into the medium and maintaining the drug concentration in a supersaturated state that corresponds to the amorphous solubility [69,71,72]. In conclusion, for amorphous polymer–phospholipid dispersions, the polymer matrix is primarily responsible for the solubility enhancement by stabilizing and shifting the amorphous solubility limit. The phospholipid, in turn, further enhances this effect, provided there is an optimal ratio of phosphatidylcholine to active compound.

Continuing with our investigation into the biopharmaceutical properties, we conducted further studies to evaluate the impact of formulations on permeability. The Parallel Artificial Membrane Permeability Assay (PAMPA) was selected to simulate passive diffusion across the gastrointestinal tract (GIT) and blood–brain barrier (BBB), which are the primary biological barriers for orally administered compounds targeting the central nervous system. The results of this experiment are presented in Table 3.

In the permeability study, similar trends to those observed in the solubility study were noted. The F7 system demonstrated the highest permeability for the active compounds, with curcumin reaching concentrations of 0.14933 mg/mL and 0.14610 mg/mL in the GIT and BBB models, respectively. Hesperetin concentrations approached 0.07258 mg/mL and 0.08901 mg/mL in the GIT and BBB models, respectively. In contrast, the F8 system exhibited the weakest permeability, with curcumin peaking at an acceptor concentration of 0.00329 mg/mL and 0.00292 mg/mL, while hesperetin reached concentrations of 0.00401 mg/mL and 0.00375 mg/mL in the GIT and BBB models, respectively.

The role of amorphization in promoting absorption is significantly greater than that of phospholipids. Increased permeation is primarily driven by the supersaturation resulting from the amorphous state rather than the solubilization capacity of the phospholipid [67]. This can be explained by the fact that the amorphous state generates free molecules that can more easily pass through biological barriers, whereas phospholipids must encapsulate the molecule to enhance absorption. Therefore, amorphization presents a simpler mechanism for promoting absorption, as it does not require additional phenomena to occur.

However, one should consider the unique role of phospholipids as excipients that enhance bioavailability. Phospholipids are endogenous compounds that exhibit biocompatibility and biodegradability. In aqueous solutions, they self-assemble into colloidal, micelle-like structures that encapsulate lipophilic compounds. Upon digestion, phospholipids stimulate the secretion of bile salts, leading to the formation of mixed micelles composed of phospholipids, bile salts, and encapsulated drug molecules. Since these structures are biocompatible, they can fuse with enterocytes, allowing the molecules to enter the cells. Inside the cells, drug-rich chylomicrons can be formed, which are then transported via the lymphatic system, bypassing the first-pass effect [34,73,74]. These effects can only be observed in in vivo studies. This phenomenon benefits the bioavailability of active pharmaceutical substances. It is important to note that an in vitro study by Fong et al. suggests that higher amounts of phospholipids do not necessarily improve permeation, while systems with lower phospholipid contents provide better permeability [56].

The main factor contributing to enhanced absorption is supersaturation, which drives passive diffusion [75]. As previously mentioned, the polymer plays a crucial role in maintaining this state. Studies on the in vivo effects of amorphous polymer dispersions show that increased solubility translates to higher plasma concentrations of the drug. Knopp et al.’s investigation demonstrated that amorphous dispersions of celecoxib enhanced dissolution rate profiles and apparent solubility in vitro, which led to improved AUC values and higher maximum serum concentrations in an in vivo rat model [76]. This indicates a correlation between biopharmaceutical studies on amorphous dispersions and pharmacokinetic experiments conducted in vivo. Furthermore, the drug-rich nanoparticles, generated in solution due to phase separation, when the amorphous solubility is exceeded, play an important role in permeation. The formed amorphous aggregates act as a reservoir, replenishing the absorbed portion of the drug, thereby maintaining supersaturation and concentration levels corresponding to amorphous solubility [58,77,78]. The critical role of drug-rich nanoparticles in bioavailability is supported by a study by Wilson et al., which showed that systems forming nanosized amorphous drug aggregates achieved higher AUC values and maximum concentrations of enzalutamide compared to systems that did not form such aggregates [79].

It is worth noting that for the formulation preparation, we chose to use PVP K30, which is recommended for the development of polymeric delivery systems for curcumin, providing superior biopharmaceutical properties. One might speculate that if another polymer had been chosen, phosphatidylcholine could have played a more significant role as a solubilizer. In this case, phospholipids could potentially be a game changer, drastically improving the solubility due to their favorable interactions with the compound. However, in our case, phosphatidylcholine had to compete with PVP K30, which shows preferential interactions with curcumin, potentially suppressing the ability of the phospholipid to improve the solubility. Additionally, the lipophilicity of the compounds must be considered. More lipophilic compounds are likely to have a higher affinity for phospholipids, which would have a more pronounced beneficial effect on the solubility. It is also important to note that one of the limitations of this research is the lack of long-term stability studies on the extrudates, which should be conducted to assess the feasibility of using these systems in pharmaceutical products.

## 4. Conclusions

In this study, we focused on obtaining amorphous PVP K30–phospholipid dispersions of curcumin and hesperetin in a fixed-dose formulation. The solubility-enhancing formulations were prepared using the hot-melt extrusion technique. In the first stage, we investigated whether factors such as the content of the active compounds, the amount of phosphatidylcholine in the carrier, or the process temperature significantly affected the achieved solubility. Our analysis indicates that the contents of the active compounds and the amount of phosphatidylcholine were statistically significant factors influencing the solubility. The second stage involved characterizing the physicochemical and biopharmaceutical properties of the formulations. Notably, the system that provided the best solubility contained 20% phosphatidylcholine. This suggests that a small addition of phospholipid interacts synergistically with the polymer, enhancing the positive effect of amorphous polymer dispersions on solubility.

Further research is needed to validate the inclusion of phospholipids in amorphous polymeric dispersions and to explore polymer–phospholipid combinations. Future studies should focus on determining how this combination affects in vivo permeability, taking into account mechanisms beyond passive diffusion that may be influenced by phospholipids. Considering the physiological fate of phospholipids, this influence could play a key role in enhancing bioavailability, a benefit that amorphous polymeric dispersions alone may not provide.

## Figures and Tables

**Table 1:** The Box–Behnken design for the three independent variables.

Name	Content of Actives (%)	Phosphatidylcholine Content in Carrier (%)	Temperature (°C)
F1	15	0	150
F2	40	0	150
F3	15	40	150
F4	40	40	150
F5	15	20	135
F6	40	20	135
F7	15	20	165
F8	40	20	165
F9	27.5	0	135
F10	27.5	40	135
F11	27.5	0	165
F12	27.5	40	165
F13	27.5	20	150
F14	27.5	20	150
F15	27.5	20	150

**Table 2:** The results of solubility studies.

		Compound			
		Curcumin		Hesperetin	
		Conc. [mg/mL]	Improv. [-fold]	Conc. [mg/mL]	Improv. [-fold]
System	Raw	0.00014 ± 0.00002	N/A	0.005 ± 0.001	N/A
	F1	5.653 ± 0.075	40,379	5.847 ± 0.085	1169
	F2	2.093 ± 0.101	14,950	2.279 ± 0.124	456
	F3	5.805 ± 0.075	41,464	6.399 ± 0.051	1280
	F4	0.310 ± 0.081	2214	0.591 ± 0.070	118
	F5	5.941 ± 0.079	42,436	6.085 ± 0.058	1217
	F6	0.246 ± 0.011	1757	0.615 ± 0.014	123
	F7	6.648 ± 0.082	47,486	7.397 ± 0.059	1479
	F8	0.192 ± 0.010	1371	0.513 ± 0.040	103
	F9	4.031 ± 0.074	28,793	3.918 ± 0.030	784
	F10	2.553 ± 0.004	18,236	2.849 ± 0.036	570
	F11	4.958 ± 0.045	35,414	5.480 ± 0.040	1096
	F12	1.423 ± 0.208	10,164	2.109 ± 0.080	422
	F13	3.145 ± 0.032	22,464	3.265 ± 0.025	653
	F14	3.160 ± 0.025	22,571	3.286 ± 0.029	657
	F15	3.200 ± 0.069	22,857	3.312 ± 0.033	662

N/A means “not applicable”.

**Table 3:** The results of permeability assay. The table includes concentrations reached in the acceptor compartment of the PAMPA models.

Assay	Formulation	Compound			
		Curcumin		Hesperetin	
		Conc. [mg/mL]	Improv. [-fold]	Conc. [mg/mL]	Improv. [-fold]
PAMPA GIT	Raw	0.00000334 ± 0.00000194	N/A	0.0000258 ± 0.00000616	N/A
	F1	0.14978 ± 0.00534	44,844	0.06318 ± 0.00189	2449
	F2	0.04483 ± 0.00170	13,422	0.01679 ± 0.00044	651
	F3	0.11888 ± 0.00652	35,593	0.06001 ± 0.00161	2326
	F4	0.00577 ± 0.00062	1728	0.00576 ± 0.00031	223
	F5	0.14316 ± 0.00550	42,862	0.06177 ± 0.00236	2394
	F6	0.00322 ± 0.00025	964	0.00423 ± 0.00032	164
	F7	0.14993 ± 0.00544	44,889	0.07258 ± 0.00097	2813
	F8	0.00329 ± 0.00027	985	0.00401 ± 0.00029	155
	F9	0.09956 ± 0.00708	29,808	0.03604 ± 0.00130	1397
	F10	0.04698 ± 0.00150	14,066	0.02109 ± 0.00123	817
	F11	0.13656 ± 0.00684	40,886	0.05555 ± 0.00165	2153
	F12	0.02250 ± 0.00070	6737	0.01839 ± 0.00084	713
	F13/14/15	0.05826 ± 0.00117	17,443	0.02591 ± 0.00034	1004
PAMPA BBB	Raw	0.0000186 ± 0.00000248	N/A	0.0000389 ± 0.0000166	N/A
	F1	0.14188 ± 0.00257	7628	0.06423 ± 0.00208	1651
	F2	0.04056 ± 0.00101	2181	0.01426 ± 0.00025	367
	F3	0.11411 ± 0.00126	6135	0.05968 ± 0.00178	1534
	F4	0.00661 ± 0.00062	355	0.00492 ± 0.00029	127
	F5	0.14497 ± 0.00292	7794	0.07770 ± 0.00196	1997
	F6	0.00284 ± 0.00025	153	0.00374 ± 0.00054	96
	F7	0.14610 ± 0.00051	7855	0.08901 ± 0.00194	2288
	F8	0.00292 ± 0.00017	157	0.00375 ± 0.00022	96
	F9	0.11223 ± 0.00170	6034	0.04205 ± 0.00158	1081
	F10	0.04440 ± 0.00090	2387	0.02047 ± 0.00118	526
	F11	0.13630 ± 0.00757	7328	0.06318 ± 0.00265	1624
	F12	0.02352 ± 0.00061	1265	0.01419 ± 0.00071	365
	F13/14/15	0.08435 ± 0.00170	4535	0.03194 ± 0.00091	821

N/A means “not applicable”.

## Data Availability

The data are contained within the article.

## Supplementary Materials

The following supporting information can be downloaded at: https://www.mdpi.com/article/10.3390/pharmaceutics17010026/s1, Table S1: The HPLC method’s validation parameters.
