# Supplementary material for: Application of the Box–Behnken Design in the Development of Amorphous PVP K30–Phosphatidylcholine Dispersions for the Co-Delivery of Curcumin and Hesperetin Prepared by Hot-Melt Extrusion
Source: Pharmaceutics. 2024 Dec 27;17(1):26. doi: 10.3390/pharmaceutics17010026 (PMC11768460; doi:10.3390/pharmaceutics17010026)
Supplement: Supplementary file 1 [file pharmaceutics-17-00026-s001.zip › pharmaceutics-3369393-supplementary.pdf]

pharmaceutics

Supplementary Materials

Application of the Box–Behnken Design in the Development of Amorphous PVP K30–Phosphatidylcholine Dispersions for the Co-Delivery of Curcumin and Hesperetin Prepared by Hot-Melt Extrusion

Kamil Wdowiak [1], Lidia Tajber [2], Andrzej Miklaszewski [3] and Judyta Cielecka-Piontek [1,*]

[1] Department of Pharmacognosy and Biomaterials, Poznan University of Medical Sciences, 3 Rokietnicka St., 60-806 Poznan, Poland; kamil.wdowiak@student.ump.edu.pl
[2] School of Pharmacy and Pharmaceutical Sciences, Trinity College Dublin, University of Dublin, D02 PN40 Dublin, Ireland; ltajber@tcd.ie
[3] Faculty of Materials Engineering and Technical Physics, Institute of Materials Science and Engineering, Poznan University of Technology, 5 M. Skłodowska-Curie Square, 60-965 Poznan, Poland; andrzej.miklaszewski@put.poznan.pl
* Correspondence: jpiontek@ump.edu.pl

Chromatographic conditions:
- Stationary phase - Dr. Maisch ReproSil-Pur Basic-C18 100 Å column, 5 µm particle size, 100 × 4.60 mm
- Mobile phase - methanol/0.1% acetic acid (80:20 v/v)
- Column temperature - 30 °C
- Flow rate – 0.6 mL/min

Table S1. The HPLC method's validation parameters.

**Curcumin**	
Parameter	Curcumin dissolved in methanol<br>Injection volume 10 µl
Linearity range (mg/mL)	0.0000208 – 0.104
Correlation coefficient (r)	0.9999
$a \pm S_a$	119606610 ± 432110
$b \pm S_b$	insignificant (α=0.05)
LOD (mg/mL)	0.0006
LOQ (mg/mL)	0.0017
Retention Time	4.136
**Hesperetin**	
Parameter	Hesperetin dissolved in methanol<br>Injection volume 10 µl
Linearity range (mg/mL)	0.000006 – 0.3
Correlation coefficient (r)	0.9999
$a \pm S_a$	55395172 ± 710539
$b \pm S_b$	insignificant (α=0.05)
LOD (mg/mL)	0.0059
LOQ (mg/mL)	0.0178
Retention Time (min)	2.651
